# Supplementary figures and images for: Integrated computational and in vivo models reveal Key Insights into macrophage behavior during bone healing
Source: PLoS Comput Biol. 2022 May 13;18(5):e1009839. doi: 10.1371/journal.pcbi.1009839 (PMC9106165; doi:10.1371/journal.pcbi.1009839)

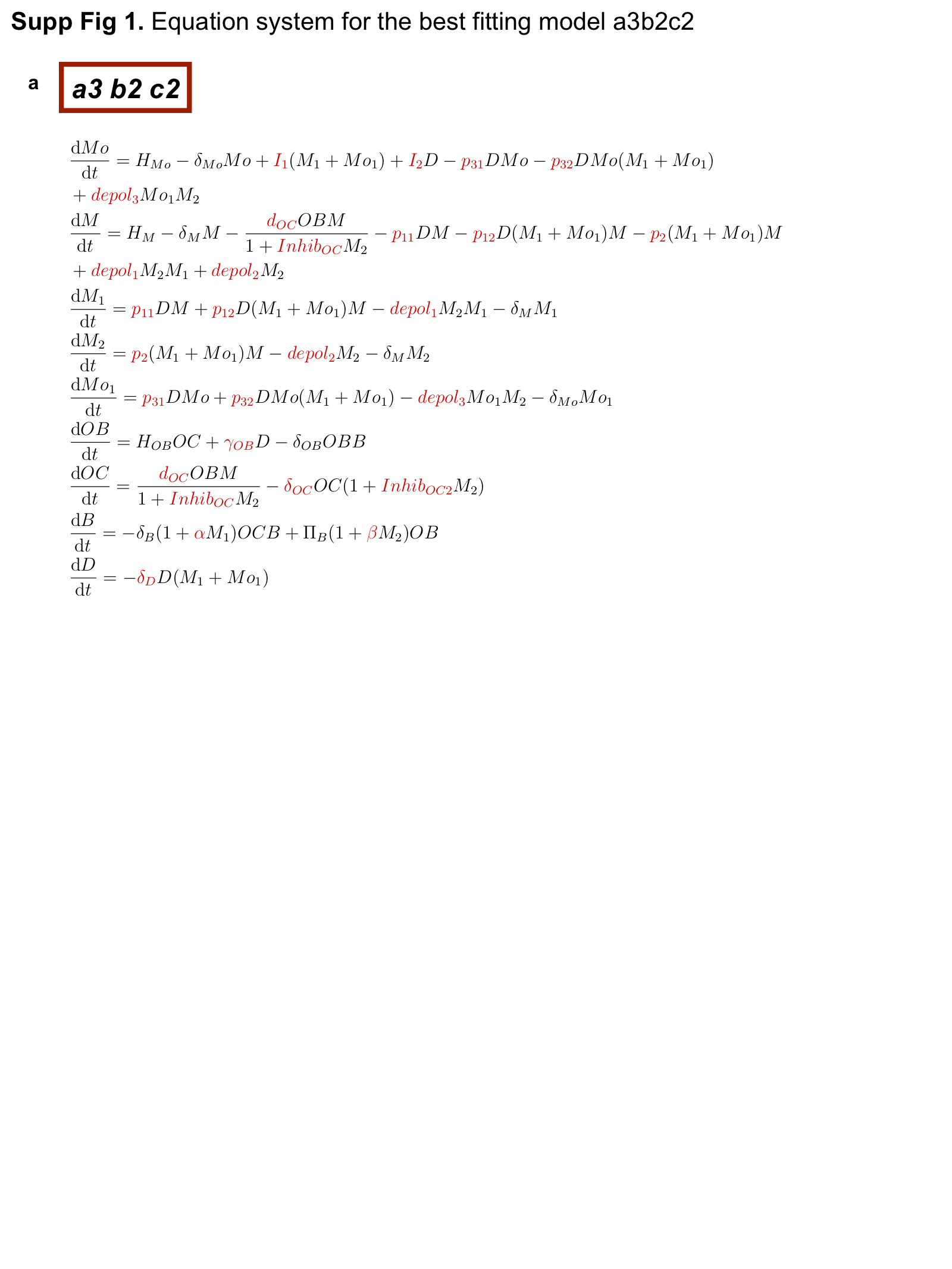

Supplement: S1 Fig — Parameters with no reference publication were estimated to obtain best possible fits to temporal dynamics data (parameters in red) and are listed in Table 3. In all equations, black terms correspond to homeostatic dynamics, whereas red terms correspond to injury dynamics. (TIFF) [file pcbi.1009839.s001.tiff]

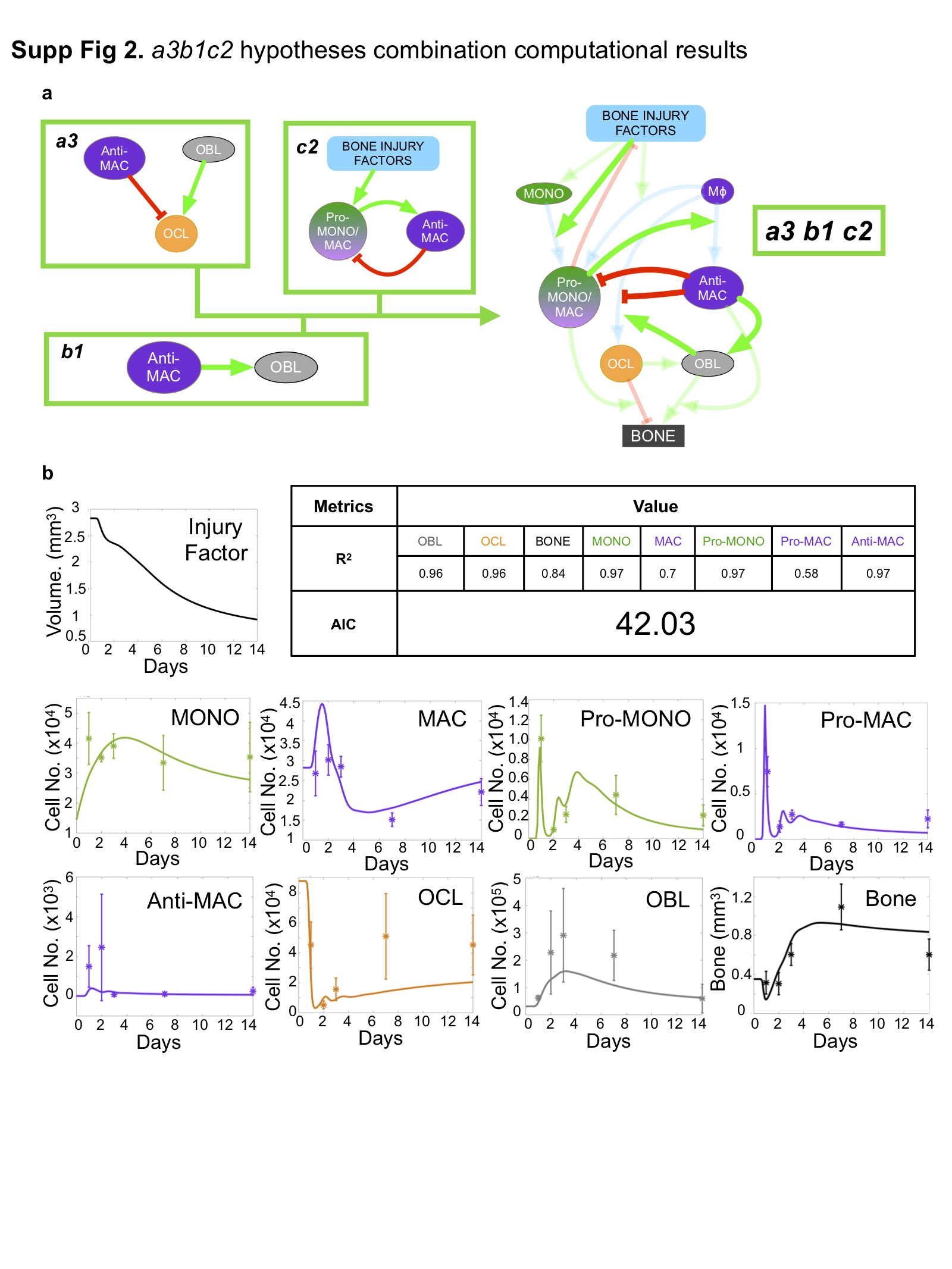

Supplement: S2 Fig — Alternative hypotheses combination a3 b1 c2 (green boxes in a-c) computational results. a Mechanism a3 assumes that osteoblasts and anti-inflammatory macrophages promote and inhibit osteoclast formation, respectively. b Mechanism b1 assumes that anti-inflammatory macrophages promote osteoblast expansion. c Mechanism c2 assumes that injury factors promote pro-inflammatory monocytes/macrophages polarization. Pro-inflammatory monocytes/macrophages promote anti-inflammatory macrophages polarization, which in return drive depolarization of pro-inflammatory monocytes/macrophages back to the naive state. d Schematic representation of the model using a3b1c2 hypothesis combination. Arrows represent positive (green) or negative (red) types of cellular interactions. e Temporal plots and corresponding goodness of fit metrics (AIC and R2s) across all populations, obtained through J∞ minimization. (TIFF) [file pcbi.1009839.s002.tiff]

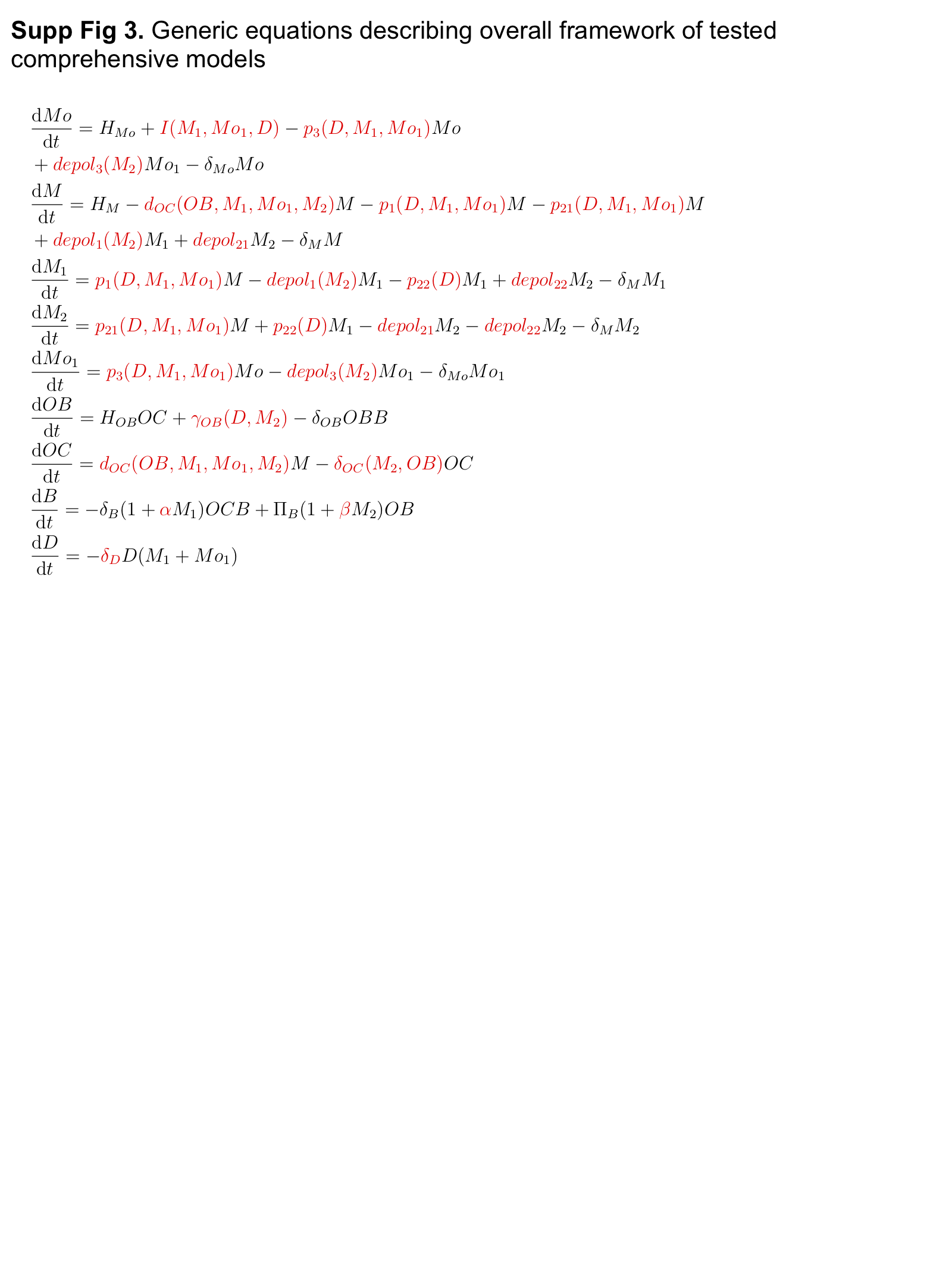

Supplement: S3 Fig — Each term (e.g formation rate, clearance, transition) is a functional form of other variables reflecting cellular interactions described in Fig 2. Black terms correspond to homeostasis, red terms correspond to injury dynamics and are described in details in Mathematical and Computational Methods. (TIFF) [file pcbi.1009839.s003.tiff]

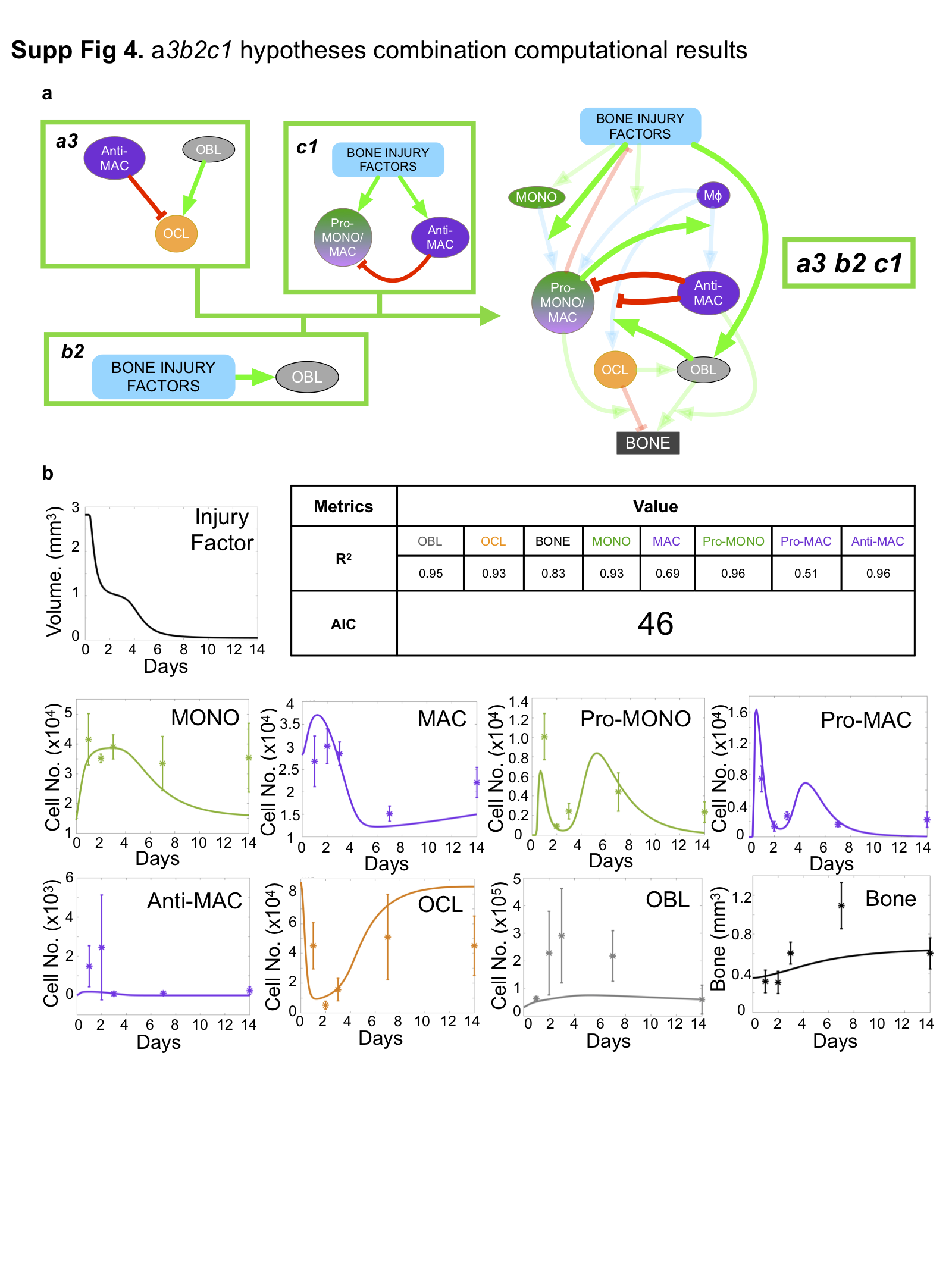

Supplement: S4 Fig — Alternative hypotheses combination a3 b2 c1 (green boxes in a-c) computational results. a Mechanism a3 assumes that osteoblasts and anti-inflammatory macrophages promote and inhibit osteoclast formation, respectively. b Mechanism b2 assumes that injury factors promote osteoblast expansion. c Mechanism c1 assumes that injury factors promote pro-inflammatory monocytes/macrophages and anti-inflammatory macrophages polarization. Anti-inflammatory macrophages drive depolarization of pro-inflammatory monocytes/macrophages back to the naive state. d Schematic representation of the model using a3b2c1 hypothesis combination. Arrows represent positive (green) or negative (red) types of cellular interactions. e Temporal plots and corresponding goodness of fit metrics (AIC and R2s) across all populations, obtained through J∞ minimization. (TIFF) [file pcbi.1009839.s004.tiff]

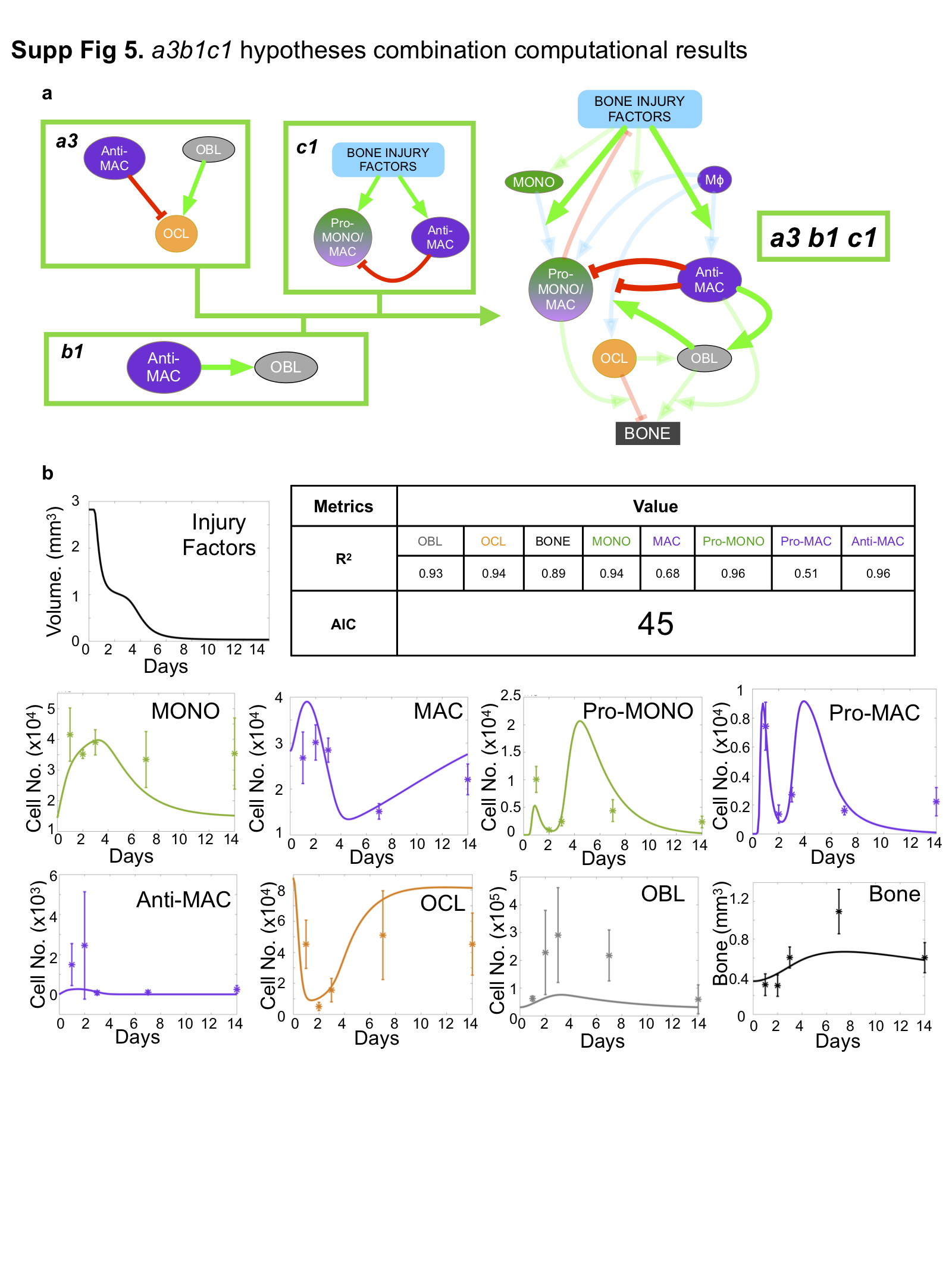

Supplement: S5 Fig — Alternative hypotheses combination a3 b1 c1 (green boxes in a-c) computational results. a Mechanism a3 assumes that osteoblasts and anti-inflammatory macrophages promote and inhibit osteoclast formation, respectively. b Mechanism b21assumes that injury factors promote osteoblast expansion. c Mechanism c1 assumes that anti-inflammatory macrophages promote pro-inflammatory monocytes/macrophages and anti-inflammatory macrophages polarization. Anti-inflammatory macrophages drive depolarization of pro-inflammatory monocytes/macrophages back to the naive state. d Schematic representation of the model using a3b2c1 hypothesis combination. Arrows represent positive (green) or negative (red) types of cellular interactions. e Temporal plots and corresponding goodness of fit metrics (AIC and R2s) across all populations, obtained through J∞ minimization. (TIFF) [file pcbi.1009839.s005.tiff]

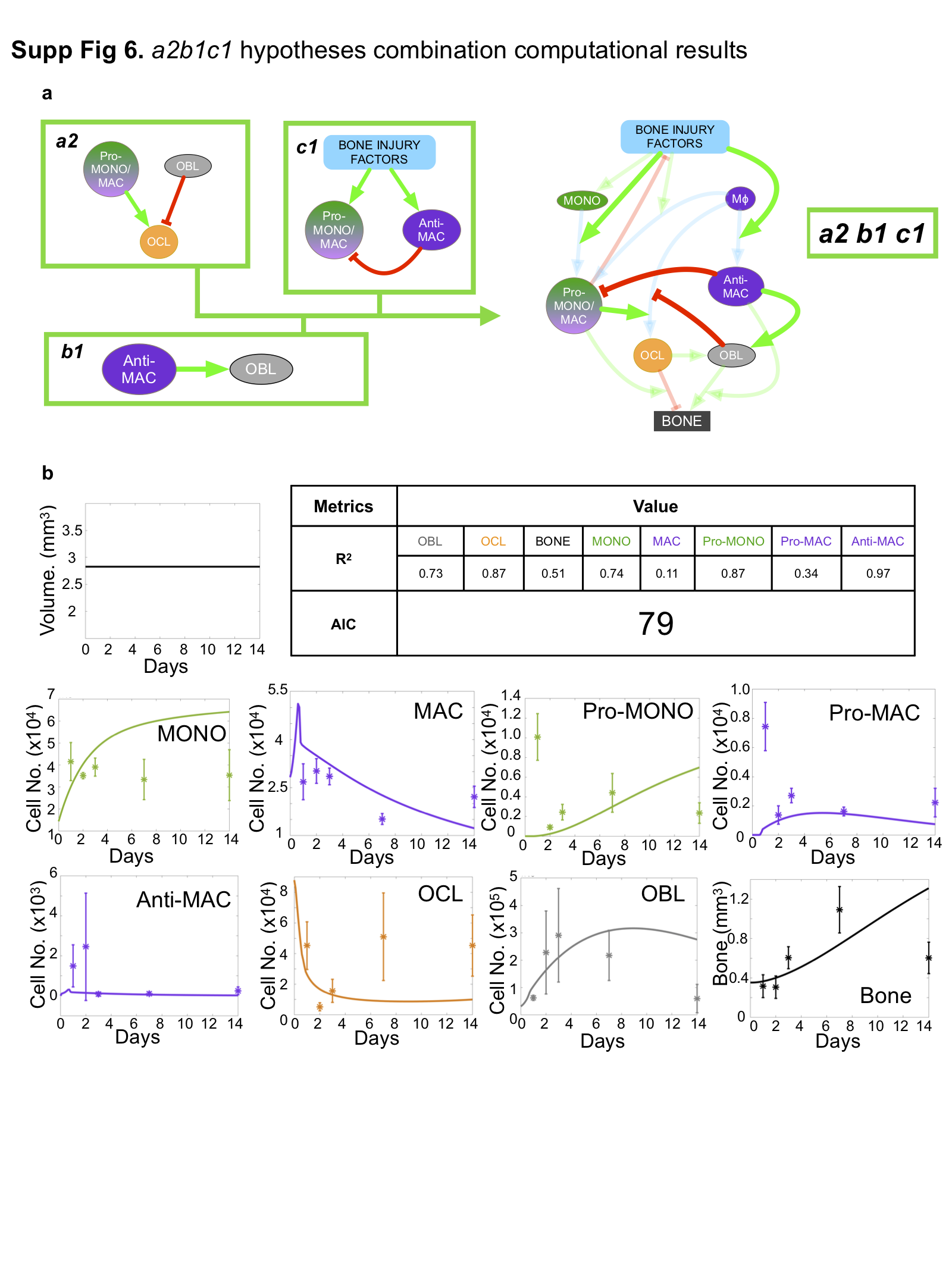

Supplement: S6 Fig — Alternative hypotheses combination a2 b1 c1 (green boxes in a-c) produces the second worst fit of all hypotheses combinations. a Mechanism a2 assumes that pro-inflammatory and macrophages and osteoblasts promote and inhibit osteoclast formation, respectively. b Mechanism b1 assumes that anti-inflammatory macrophages promote osteoblast expansion. c Mechanism c1 assumes that injury factors promote pro-inflammatory monocytes/macrophages polarization and anti-inflammatory macrophages. The latter drive depolarization of pro-inflammatory monocytes/macrophages back to the naive state. d Schematic representation of the model using a2b1c1 hypothesis combination. Arrows represent positive (green) or negative (red) types of cellular interactions. e Temporal plots and corresponding goodness of fit metrics (AIC and R2s) across all populations, obtained through J∞ minimization. (TIFF) [file pcbi.1009839.s006.tiff]
